# Influenza in feral cat populations: insights from a study in North-East Italy

**DOI:** 10.3389/fvets.2024.1439354

**Published:** 2024-08-23

**Authors:** Lara Cavicchio, Mery Campalto, Marilena Carrino, Laura Lucchese, Letizia Ceglie, Alice Fincato, Lorenza Boscolo Cegion, Elisa Mazzotta, Maria Serena Beato, Alda Natale

**Affiliations:** Istituto Zooprofilattico Sperimentale delle Venezie (IZSVe), Legnaro, Italy

**Keywords:** influenza, cat, Italy, companion animals' shelters, feline colony

## Abstract

Influenza A virus (IAV) can cause high morbidity and mortality in domestic and wild avian species and it is able to infect mammals as well. IAV in cats is sporadic and self-limiting but the recent findings of high pathogenicity avian influenza virus (HPAIV) with genetic signatures of mammalian adaptation, in domestic cats, has raised new concerns about the potential role of cats in the virus ecology. The present study aimed to investigate the circulation of IAV in companion animals' shelters in North-eastern Italy. All samples were collected from feral cats living in feline colonies that were hosted in the companion animals' shelters for the requisite period to administer the veterinary treatments. Between 2021 and 2022, 389 oropharyngeal swabs and 279 sera were collected. All swabs tested negative for IAV and the only one ELISA positive serum sample resulted H5 positive by HI test with a titer of 1:80. Despite the sporadic occurrence of influenza in cats, continuous monitoring is crucial due to the evolving zoonotic nature of the virus.

## 1 Introduction

Influenza viruses are negative-sense, single stranded segmented RNA viruses, belonging to the *Orthomyxoviridae* family. This virus family comprises four genera: A, B, C, and D (1–3). In humans, influenza type A and B viruses are responsible for acute respiratory diseases, but while type B primarily circulates among humans, type A viruses (IAV) have been detected in a variety of animal species including bird and mammals (1, 4, 5).

Influenza A viruses are categorized into different subtypes based on the antigenic profile of the two major surface glycoproteins: the haemagglutinin (HA) and neuraminidase (NA) (3, 6). Influenza are rapidly evolving viruses, whose evolution is driven by the so called “antigenic drift,” a constant accumulation of point mutations primarily affecting the HA gene, and “antigenic shift,” resulting from genetic reassortment events among different influenza viral strains originating even from different animal species (1, 7).

Each IAV subtype commonly exhibits restricted host range. However, there are instances where strains belonging to a subtype specific to one host species may be transmitted to another species. Wild aquatic migratory birds are considered to be the natural reservoirs for these viruses, and spill over events into other animal hosts such as horses, cats, dogs, and swine can occur (8–11).

IAV disease in cats is infrequent and typically self-limiting, however, pathogenic strains may cause a severe disease, resulting in high mortality. There are four described IAV subtypes mainly associated with acute respiratory syndrome in cats: canine influenza H3N2, low pathogenicity avian influenza virus (LPAIV) H7N2, the pandemic H1N1 swine-origin and the high pathogenicity avian influenza virus (HPAIV) H5N1 (12).

The HPAI H5N1 virus has caused significant losses to poultry flocks and wild bird populations and there is a growing concern as an increasing number of mammalian species are becoming susceptible to H5N1 virus. From 2003 to 2023, more than ten cases of IAV infections, involving felines were reported worldwide (13, 14). It is noteworthy that in six of these cases, the feed containing raw chicken meat was suspected to be the source of the infection with H5N1 virus (13–21). Recently, in Europe two cases of HPAI H5N1 infections were reported, in domestic cats exhibiting respiratory and neurological signs. In both cases, in France in December 2022 and in Poland in 2023, the involved IAV strain showed genetic signatures of mammalian adaptation (22, 23). Furthermore, in April 2023, a cat in Italy, living in a rural farm with H5N1 HPAIV-positive hens, seroconverted without exhibiting clinical signs (24). In late March and early April 2024, Texas reported detection of HPAI H5N1 in several cats from several dairy farms that had experienced HPAI H5N1 virus infections in dairy cows (25). This suggests that the virus may have spread to the cats either from the affected dairy cows, raw cow milk, or from wild birds associated with those farms. These recent cases have raised concerns about the potential role of cats in the virus ecology.

In 2006, a H3N2 strain, known as canine influenza, emerged in Southeast Asia, and rapidly spread in the region, becoming endemic before reaching the USA and Canada (12, 26–28). Subsequently, H3N2 outbreaks were observed among cats, characterized by fever, sneezing, coughing, respiratory distress, and lethargy. Although cats can contract the virus through direct transmission from dogs or from other infected cats, it replicates less effectively in cats compared to dogs (29). Moreover, natural outbreaks among felines are infrequent and mainly occurred within shelter environments (29).

Several studies suggest that in 2009, cats globally became infected with the pandemic H1N1 swine origin influenza virus, probably due to a direct transmission from their owners. In Italy, it was reported an outbreak of respiratory and gastrointestinal disease in a colony of 90 cats, leading to 25 fatalities (12, 30).

The first detection of H7N2 in cats was reported in the USA in December 2016 (29, 31). The virus was closely related to strains that circulated in poultry markets in the USA between 1994 and 2006. Subsequently, the virus spread into several cat-shelters, and during the outbreaks, a veterinarian became infected with the H7N2 virus of feline origin, exhibiting respiratory symptoms, and serological evidence of H7N2 infection was identified in a shelter worker (29, 31).

The present study was part of a broader investigation on zoonotic pathogens in cats at companion animals' shelters in North-eastern Italy, that investigated the circulation of *Capnocytophaga* spp., *Bartonella* spp., Norovirus, Rotavirus A, Cowpoxvirus, Mammalian Orthoreovirus (MRV), Hepatitis E virus (32), and SARS-CoV-2 (33), including IAV in feral cats.

The companion animals' shelters that participated in the present study, hosted feral cats, primarily originating from different feline colonies, for the required period to deliver veterinary care and treatments. During this period, workers (veterinarians and volunteers) were potentially exposed to zoonotic pathogens due to the unknown infection status of the hosted cats. Consequently, companion animals' shelters were identified as a potential hotspot for the transmission of zoonotic pathogens due to the high turnover of feral cats and frequent contacts with volunteers, who often lacked knowledge about biosecurity and zoonotic risks.

Furthermore, the cat colonies from which cats were sampled are in an HPAIV epidemic area in 2021–2022. It is plausible to assume that feral cats may have been exposed to H5N1 positive birds during this period. Although literature documented low prevalence of HPAI H5N1 antibodies in cat sera even in areas where birds tested positive for HPAI H5N1 virus, the monitoring is crucial. This becomes even more important following recent cases of HPAI H5N1 in cats.

## 2 Material and method

### 2.1 Samples and sampling area

**Figure 1:**
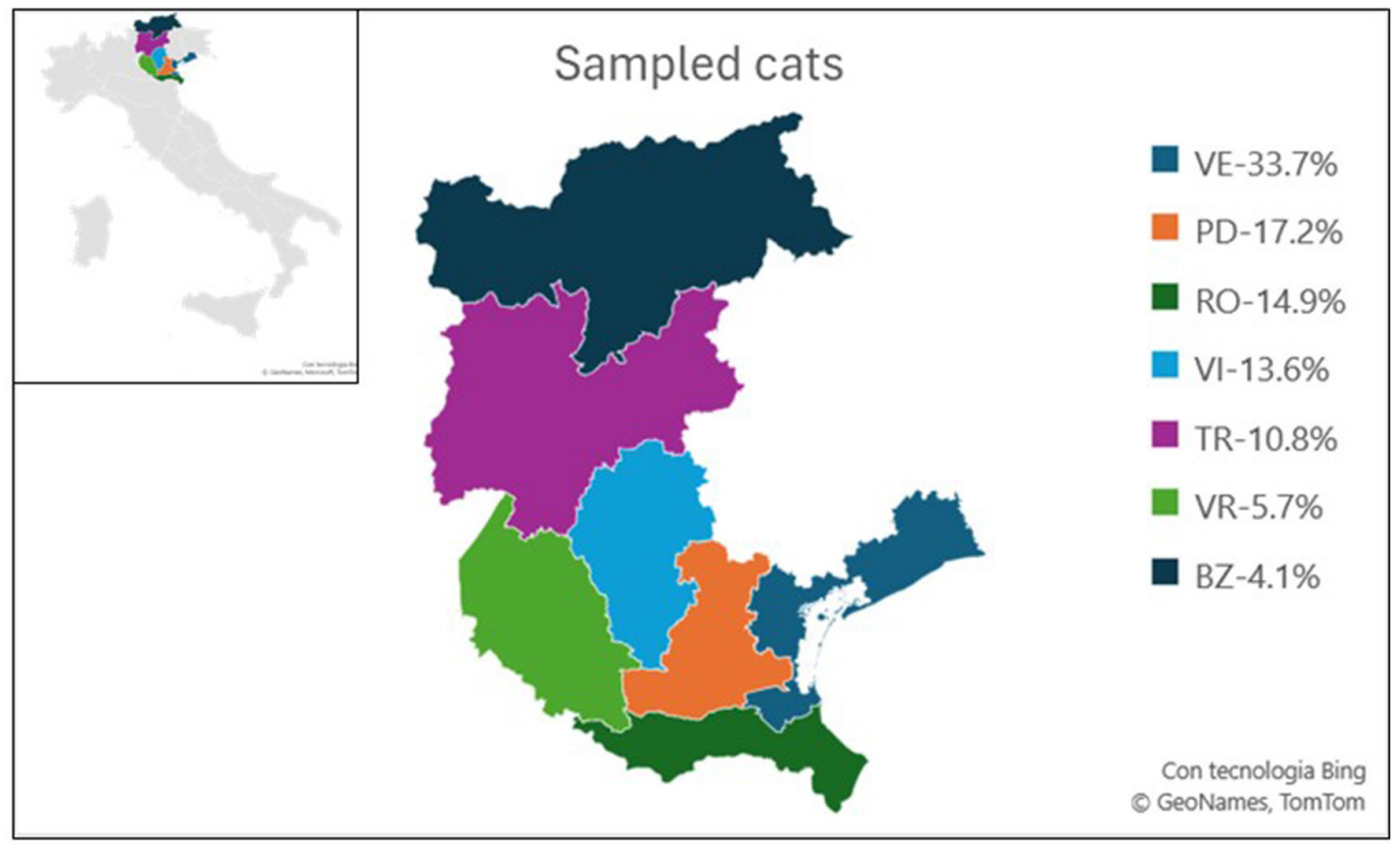
Map illustrating the sampling zones of the study. Each province is color-coded, with the percentage of sampled animals from each province relative to the total sampled in the study indicated. Veneto provinces: Venice (VE), Padua (PD), Rovigo (RO), Vicenza (VI), Verona (VR), Trentino-Alto Adige provinces: Trento (TR), and Bozen (BZ).

Oropharyngeal (OP) swabs and sera were collected from eight different companion animals' shelters located in seven provinces, within Veneto and Trentino Alto Adige regions in North-eastern Italy, from May 2021 to June 2022 (Figure 1). A sample size of 385 animals was considered necessary to estimate prevalence with a 95% confidence interval, assuming an expected prevalence of 50% (with no prior information about the population). For each cat were collected one OP swab and one serum and each animal was sampled only once. Given the nature of the animals—feral cats from colonies housed in shelters only for medical treatment and then released—it was not possible to sample animals more than once. Additionally, an oropharyngeal swab was selected as considered less invasive than a nasal swab. OP swabs collected in 2021 were examined individually, whereas OP swabs collected in 2022 were analyzed in pools of 10 samples each, to optimize resources. Pooled samples originated from the same province. Each swab was rehydrated with 1 mL of Minimal Essential Medium (Sigma Aldrich, Burlington, Massachusetts, USA) supplemented with antibiotics, albumin 0.5% v/v, HEPES buffer. Each sample was stored at −80°C until use. All samples were collected by veterinarians for diagnostic, therapeutic, or prophylactic purposes adhered to the Guide for the Care and Use of Laboratory Animals and Directive 2010/63/EU for animal experiments (National law: D.L. 26/2014), with ethical committee approval received for the study (CE_IZSVE 8/2020).

### 2.2 RNA extraction and RT real-time PCR screening

Viral RNA was extracted from 100 μL of each sample using a semi-automated method on the KingFisher™ Flex Purification System (Thermo Fisher Scientific, Waltham, MA, USA), with the ID Gene™ Mag Universal Extraction Kit (Innovative Diagnostics, Grabels, France). Additionally, to assess RNA extraction efficiency and validate each negative result, a universal heterologous RNA control, Intype IC-RNA (Indical Bioscience GmbH, Leipzig, Germany) was added to each sample during the extraction phase at a 1:10 ratio of the total elution volume and subsequently co-amplified using the primers and probe indicated by Hoffman et al. (34). Extracted viral RNA was stored at −80°C until use. Extracted RNA samples were tested for IAV using a one-step Real Time RT-PCR targeting the conserved M gene of IAV of swine origin (35), performed on a CFX 96 Deep Well Real time PCR system (Bio-Rad Laboratories Inc., Hercules, CA, USA) with the AgPath-ID™ One-Step RT-PCR (Applied Biosystems, Thermo Fisher Scientific, Waltham, MA, USA). In detail, the reaction was carried out in 25 μL volume consisting of 2.5 μL of water, 12.5 μL of 2X RT-PCR AgPath-ID buffer, 2 μL of premix of the target primers IAV and 2 μL premix of internal control (IC) primers EGFP as described in Hoffman et al. (35), 1 μL enzyme mix, 5 μL of the extracted RNA. Real time RT-PCR reactions were run using the following thermal profile: retro-transcription at 45°C for 10 min; a Taq activation step at 95°C for 10 min followed by 42 cycles with denaturation at 95°C for 15 s, annealing at 55°C for 20 s, extension at 72°C for 30 s.

In light of the method's capability to identify influenza strains circulating in swine, it is therefore able to identify the pandemic strain currently circulating among humans as well. The *in silico* analysis conducted using all feline and canine influenza sequence available in GenBank (https://www.ncbi.nlm.nih.gov/genbank/), last accessed July 2020) showed a correct alignment of primers and probes used with all the eight sequence (Supplementary material 1). Method proved to be capable to detect the canine influenza strain available in the laboratory: A/canine/Korea/LBM412/08. Moreover, during the validation of the assay, one H5N1 avian strain were included: H5N1/chicken/3401/05, and the method *in silico* can detect HPAI H5N1 strains, circulating in Italy between January 2021 and December 2022 (Supplementary material 2). Furthermore, during the validation tests, the method showed to accurately detect samples positive for influenza when processed in pools of up to 10 samples per pool. The limit of detection (LoD) of the method, established during the validation process, was equal to 10^0.4^TCID_50_/100 μl with a 96.3% of agreement between different replicates in different runs (data not shown).

### 2.3 Serological analysis

The commercial ELISA kit Influenza A Virus Antibody Test Kit (ELISA1 - IDEXX, Westbrook, Maine, USA), with IAV antigen coated on the wells, was used as screening to analyse the serum samples. Samples positive to ELISA1 were tested in series by another commercial ELISA kit (ELISA2) ID Screen^®^ Influenza A Antibody Competition Multi-species (IDVet, Grabels, France) with NP of IAV coated on the wells, was used to assess positive samples. Both the ELISA kits were used following the manufacturer instructions. The positivity cut-off for ELISA1 was S/N <0.60, for ELISA2 S/N ≤ 0.45. Furthermore, positive samples were assessed by haemagglutination inhibition test (HI) using the antigens selected according to available literature on IAV infections in cats (24, 36, 37) and namely: an H5 (H5N1 HPAI A/turkey/Italy/21VIR9520-3/2021), H7 (H7N1 A/Africa starling/England/983/79), H3 (H3N8 A/psitt/Italy/2873/00; H3N2 A/sw/It/312583/2009), and H1 (H1N1 A/swine/Italy/711/06; H1N2 A/swine/Italy/4159/06; H1N1 A/Verona/Italy/2810/09 human pandemic). Specifically, three volumes of receptor-destroying enzyme (RDE Seiken) were added to one volume of serum. The mixture was incubated overnight at +37°C, and subsequently inactivated at +56°C for 30 min and brought to a final dilution of 1:10 (v/v) adding six volumes of PBS. To remove non-specific haemagglutinating factors, one volume of chicken erythrocytes was added to 10 volumes of serum and incubated at +4°C under gentle shaking for 1 h, before removing erythrocytes by centrifugation at 2,000 rpm for 10 min. HI tests were performed using four haemagglutinating units of virus with 0.5% chicken erythrocytes according to standard procedures. Two-fold dilutions were tested with the first dilution tested being 1:20. The HI tire was expressed as the last dilution at which complete HI was observed.

## 3 Results

**Figure 2:**
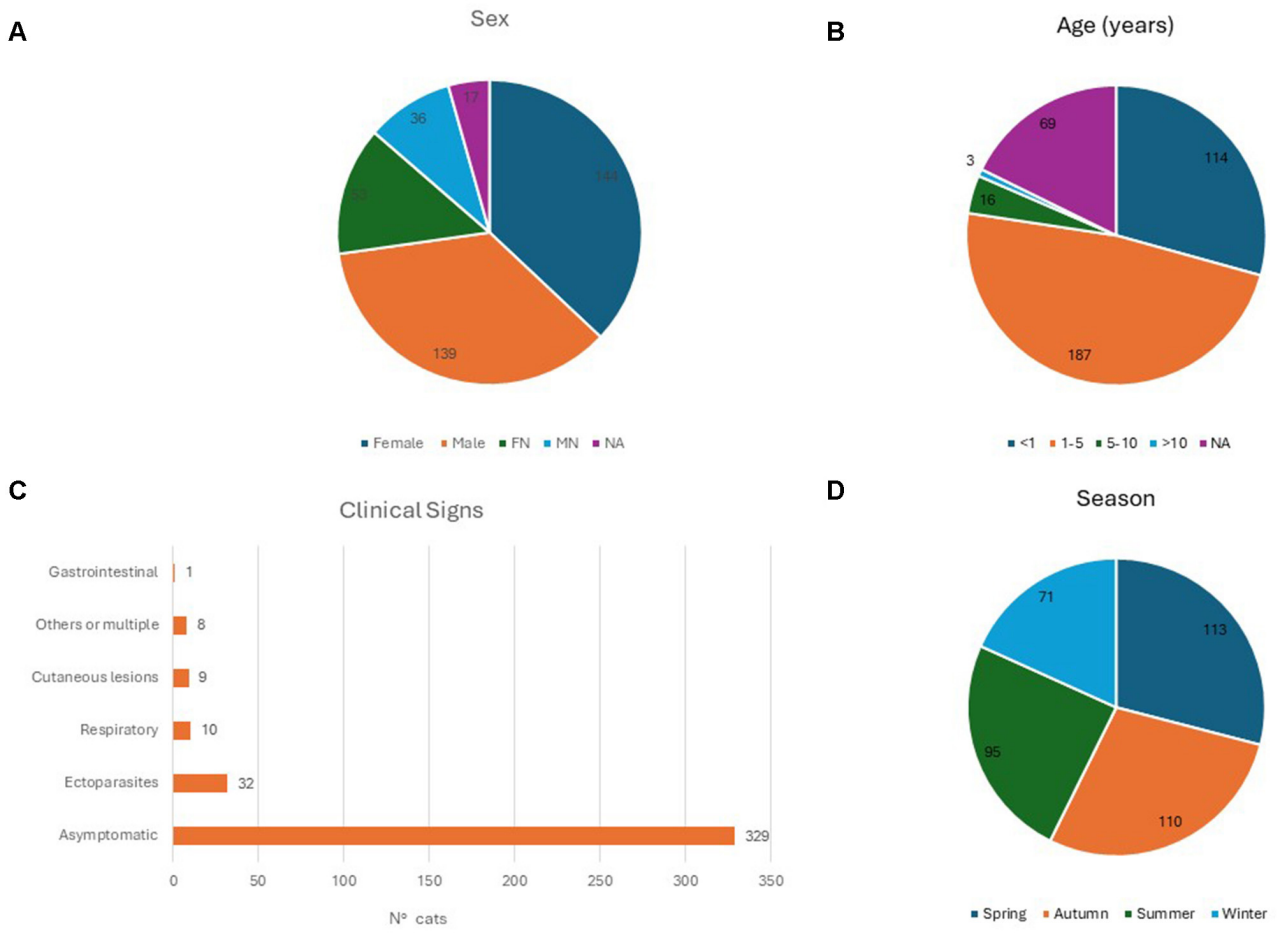
Comprehensive analysis of sampled cats. **(A)** Pie chart depicting the distribution of sampled cats by sex (FN, female neutered; MN, male neutered; NA, not available); **(B)** age distribution of sampled cats in years; **(C)** histogram categorizing cats based on clinical symptoms; **(D)** pie chart illustrating the distribution of sampled cats by season of sampling.

During the study period, OP swabs were collected from each of the 389 enrolled cats. One hundred and ninety-three samples were collected in 2021 and 196 in 2022. All sampled cats were European shorthair and their anamnestic information are resumed in Figure 2. More than half of the samples were collected during the warmer seasons, spring and summer, respectively 29.1% and 24.4 %. Samples were distributed in Veneto region in five provinces: Venice (*n* = 131, 33.7%), Padua (*n* = 67, 17.2%), Rovigo (*n* = 58, 14.9%), Vicenza (*n* = 53, 13.6%), Verona (*n* = 22, 5.7%), and in two provinces of Trentino Alto Adige: Trento (*n* = 42, 10.8%) and Bozen (*n* = 16, 4.1%) (Figure 1). Most sampled cats were young subjects: (*n* = 187 between one and five y/o and *n* = 114 were <1 y/o). Nineteen subjects were adult/senior cats (5–10 y/o, and >10 y/o), while for 69 cats this information was not available. Three hundred and twenty-nine animals were asymptomatic, ten showed respiratory signs, one exhibited gastrointestinal signs, 32 animals were found to have ectoparasites, nine animals showed skin lesions. Additionally, eight animals presented with more than one clinical signs. All the OP swabs tested negative for IAV screening by Real Time RT-PCR.

The sera were available only for 279 out of 389 cats. The serology performed with the screening ELISA1 on the 279 cat sera yielded a positive result for one sample (0.19 S/N) with an overall prevalence of 0.36% (*n* = 1/279). This sample was confirmed positive with ELISA2 (0.06 S/N) and by HI test with a titer of 1:80 for the H5 antigen. All other antigens (H7, H3, and H1) gave negative result. The positive sample originated from the Veneto region and pertained to a five-and-a-half-year-old spayed female cat that exhibited dermatological symptoms (diffuse alopecia) at the time of collection.

## 4 Discussion

The present investigation was part of a wider research project conducted between 2021 and 2022 with the aim of determining the circulation of zoonotic and/or potentially zoonotic microorganism in health-shelters, considered as hot spot of animal-human transmission of microorganisms. The project included influenza viruses among the zoonotic agents and the present papers present results of a 13-month surveillance in eight health shelters located in North-eastern Italy.

The tested health shelters were located in a densely populated poultry area (DPPA) with recurrent incursion of avian influenza infections by the H5N1 HPAI in wild and domestic avian species. In particular, during the study period, HPAI H5N1 epidemic occurred with 263 outbreaks in domestic poultry and at least 24 cases in wild avian species (38). The circulation of avian influenza during these epidemic outbreaks in colony cats, which potentially have been exposed to positive HPAI H5N1 influenza wild birds, is of significant interest. Our results suggested that the risk of exposure to IAV among the stray cats' population is low. The studied population did not report significant prevalences for IAV both in molecular and serological analyses, confirming the previous reports (29). Notwithstanding the assertion that close contact between companion animals and humans plays a significant role in the epidemiology of IAV (39, 40) in this study possibly the interaction between free-roaming, stray cats and humans is minimal.

In addition, regarding the potential transmission of IAV from humans to cats at companion animals' shelters, our results showed no detection of the H1N1 pandemic strain (H1pdmN1pdm). This finding may be attributed, at least in part, to the widespread adoption of personal protective equipment such as masks and gloves in the post-pandemic period, which greatly limited influenza virus circulation in humans. In addition, over 50% of the samples were collected during summer and spring, when the spread of influenza in humans is typically limited.

The available literature indicates that the majority of influenza outbreaks in cats and instances of transmission to humans have occurred in animal shelters, which are characterized by high population density (12, 29). However, all cats housed in the shelters under study, tested negative for the pandemic strain as well, confirming the effective management of the facilities.

In conclusion, our data indicate that influenza in cats remains a sporadic infection, with the transmission of IAV from avian species to cats occurring only rarely.

Nevertheless, in light of the progressive mutation of IAV and the potential for its spread to wild and domestic mammals, it is imperative to maintain surveillance programs on these species, as already implemented by several European Union member states (41). Feral cats, due to their ethological intermediate position between domestic and wild, and their frequent contact with humans, represent an optimal epidemiological sentinel for the risk of IAV mutations and spread. Passive surveillance on this species is also more feasible than the one on wildlife.

## Data availability statement

The original contributions presented in the study are included in the article/Supplementary material, further inquiries can be directed to lcavicchio@izsvenezie.it.

## Ethics statement

The animal study was approved by the Ethical Committee of the Istituto Zooprofilattico Sperimentale delle Venezie - CE_IZSVE 8/2020. The study was conducted in accordance with the local legislation and institutional requirements.

## Author contributions

LCa: Investigation, Writing – original draft, Writing – review & editing. MCam: Investigation, Writing – review & editing. MCar: Investigation, Writing – review & editing. LL: Project administration, Writing – review & editing. LCe: Resources, Validation, Writing – review & editing. AF: Investigation, Writing – review & editing. LBC: Investigation, Writing – review & editing. EM: Conceptualization, Project administration, Supervision, Visualization, Writing – original draft, Writing – review & editing. MB: Conceptualization, Writing – original draft, Writing – review & editing. AN: Conceptualization, Funding acquisition, Supervision, Writing – review & editing.
